# Pulse Index Continuous Cardiac Output-Guided Hemodynamic Therapy in Living Donor Kidney Transplantation: A Prospective Cohort Study

**DOI:** 10.14740/jocmr6632

**Published:** 2026-07-31

**Authors:** Van Dung Le, Huu Tu Nguyen, Quang Thuy Luu, Minh Hoang Thach, Thi Thuy Trang Tran, Van Dong Pham, Francis Zamora, Gary TA-Wei Yu, Viet Hoang Dinh

**Affiliations:** a Department of Anesthesiology and Intensive Care, Cho Ray Hospital, Ho Chi Minh City, Vietnam; b Hanoi Medical University, Hanoi, Vietnam; c Center of Anesthesia & Surgical Intensive Care, Viet Duc Hospital, Hanoi, Vietnam; d Center for Health Disparities and Molecular Medicine, Loma Linda University, Loma Linda, California, USA

**Keywords:** Kidney transplantation, Goal-directed hemodynamic therapy, PiCCO, Stroke volume variation, Delayed graft function, Transpulmonary thermodilution, Perioperative fluid management

## Abstract

**Background:**

Central venous pressure (CVP)-guided fluid therapy remains common during kidney transplantation, although CVP is a static marker with limited ability to predict fluid responsiveness. This prospective observational cohort study evaluated whether pulse index continuous cardiac output-guided goal-directed hemodynamic therapy (GDHT), using dynamic and volumetric hemodynamic variables, was associated with improved early graft function compared with conventional CVP-guided management in living donor kidney transplantation.

**Methods:**

Eighty living donor kidney transplant recipients at Cho Ray Hospital, Vietnam, were enrolled between May 2024 and December 2025. Patients were managed according to the institutional anesthetic monitoring protocol in use at the time of surgery: CVP-guided fluid therapy (group C, n = 40) or PiCCO-guided GDHT (group P, n = 40). The PiCCO protocol incorporated stroke volume variation, pulse pressure variation, cardiac index, global end-diastolic volume index, systemic vascular resistance index, and extravascular lung water index. Outcomes included intraoperative fluid volume, vasopressor use, postoperative urine output, serum creatinine through postoperative day 7, delayed graft function, and postoperative complications.

**Results:**

Baseline recipient, donor, and operative characteristics were comparable between groups, with the exception of cold ischemia time, which was longer in group P. Total intraoperative fluid volume was lower in group P than in group C (2,656 ± 480 mL vs. 3,097 ± 555 mL; P = 0.001), and vasopressor use was numerically lower but not statistically significant (12.5% vs. 27.5%; Fisher’s exact P = 0.161). First-hour postoperative urine output was higher in group P (1,235 ± 162 mL vs. 1,010 ± 315 mL; P < 0.001). Serum creatinine was similar on postoperative day 1 but was significantly lower in group P on day 3 (1.3 (0.8–1.8) mg/dL vs. 1.6 (1.4–1.9) mg/dL; P = 0.036); the difference on day 7 was not statistically significant (1.1 (0.7–1.4) mg/dL vs. 1.3 (1.1–2.1) mg/dL; P = 0.16). No patient in either group developed delayed graft function within 7 days. Extravascular lung water index remained within the reference range during surgery, and no patient developed clinical pulmonary edema.

**Conclusions:**

In this prospective cohort, PiCCO-guided GDHT was associated with lower intraoperative fluid administration, numerically lower vasopressor use, and faster early recovery of graft function after living donor kidney transplantation. These findings support further evaluation of multiparameter hemodynamic monitoring as an individualized perioperative strategy in kidney transplantation.

## Introduction

Chronic kidney disease (CKD) and end-stage renal disease (ESRD) constitute a mounting global health crisis, affecting more than 10% of the world’s population and exceeding 800 million individuals worldwide [1]. For patients with ESRD, kidney transplantation remains the renal replacement therapy and is associated with substantially better survival than remaining on dialysis or on a transplant waiting list [2]. Transplantation rates have risen progressively across Europe and North America [3], yet the disparity between organ supply and demand remains substantial. In low- and middle-income countries (LMICs), including those of Southeast Asia, access to transplantation is further constrained by inadequate infrastructure, limited financial resources, and inequities in donor availability [4]. In this context, optimizing perioperative management for each available graft is clinically important.

The perioperative period is a potentially modifiable determinant of early graft function. Ischemia-reperfusion injury is unavoidable during kidney transplantation and contributes to delayed graft function (DGF) [5]. DGF has been associated with increased risks of acute rejection, chronic graft loss, and mortality [6–8]. Intraoperative hemodynamic management is therefore a key target for intervention because both inadequate perfusion and excessive venous congestion may impair graft recovery.

For decades, perioperative fluid therapy in kidney transplantation has commonly been guided by central venous pressure (CVP) [9]. However, CVP is a static marker of filling pressure and is affected by ventricular compliance, valvular disease, intrathoracic pressure, venous tone, and chronic cardiovascular comorbidity. Systematic evaluations have shown that CVP has poor predictive value for fluid responsiveness, with performance close to chance [10, 11]. In mechanically ventilated patients, dynamic indices such as pulse pressure variation (PPV) and stroke volume variation (SVV) predict fluid responsiveness more accurately than CVP [12, 13]. In kidney transplantation, higher intraoperative CVP has also been associated with slow or delayed graft function, suggesting that CVP-driven volume loading may be harmful in some patients [14].

Goal-directed hemodynamic therapy (GDHT) uses dynamic hemodynamic variables to individualize fluid and vasoactive drug administration. GDHT has been associated with reduced postoperative acute kidney injury and other complications in major surgery [15–17]. In kidney transplantation, systematic reviews and individual cohort studies suggest that goal-directed approaches may improve perioperative fluid balance and early clinical outcomes, although the evidence base remains limited and heterogeneous [18–20].

The pulse index continuous cardiac output (PiCCO) system combines transpulmonary thermodilution with arterial pulse contour analysis and provides SVV, PPV, cardiac index (CI), global end-diastolic volume index (GEDVI), systemic vascular resistance index (SVRI), and extravascular lung water index (EVLWI). This multiparameter profile is particularly relevant for patients with ESRD, who may be vulnerable to both under-resuscitation and iatrogenic pulmonary congestion [21]. Despite the substantial evidence base for GDHT in perioperative medicine, data on PiCCO-guided management specifically in living donor kidney transplantation remain sparse, and evidence from Southeast Asian transplant centers is particularly limited. We therefore conducted a prospective observational cohort study comparing PiCCO-guided GDHT with conventional CVP-guided fluid therapy in living donor kidney transplant recipients, focusing on intraoperative fluid administration, hemodynamic stability, vasopressor use, and early graft function.

## Materials and Methods

### Study design and ethics

This prospective observational cohort study included two comparison groups and was conducted at Cho Ray Hospital, Ho Chi Minh City, Vietnam, between May 2024 and December 2025. The study protocol was approved by the Cho Ray Hospital Ethics Committee (Approval No. 1794/CN-HDDD, dated May 9, 2024) and conducted in accordance with the Declaration of Helsinki. Written informed consent was obtained from all participants prior to enrolment.

### Patient population

Eligible participants were adult patients (aged ≥18 years) scheduled for elective living donor kidney transplantation, with American Society of Anesthesiologists (ASA) physical status class III or IV, who provided informed consent. Exclusion criteria included: receipt of a deceased-donor organ, pre-existing severe cardiac dysfunction (left ventricular ejection fraction < 40%), cardiac arrhythmia precluding reliable SVV/PPV interpretation, contraindication to femoral arterial catheterization (peripheral arterial disease, anatomical abnormality), pre-existing pulmonary edema, coagulation disorders, and inability to provide informed consent.

Parathyroid hormone (PTH), serum calcium, and serum phosphorus were assessed as part of routine pre-transplant evaluation, and patients found to have a parathyroid adenoma underwent parathyroidectomy before transplantation.

### Group assignment

Patients were consecutively managed according to the anesthetic hemodynamic monitoring protocol applied at the time of their procedure. Group C received conventional CVP-guided fluid therapy, whereas group P received PiCCO-guided GDHT. Group assignment was not randomized and reflected institutional logistics rather than patient characteristics. The PiCCO monitor was fixed in a single recipient operating room, so allocation was determined by operating room scheduling under the institutional protocol rather than by patient factors. There was no randomization, no allocation concealment, and treating anesthesiologists could not be blinded to the monitoring strategy; outcome assessors and data analysts were blinded to group assignment.

### Sample size calculation

The planned sample size was informed by prior studies comparing dynamic parameter-guided fluid management with conventional or liberal strategies in kidney transplantation [22, 23]. Assuming 80% power and a two-sided alpha level of 0.05, a minimum of 43–48 participants per group was estimated to be required. Allowing for an anticipated dropout rate of approximately 10%, 100 patients were planned for enrollment. Because of constraints related to the living donor transplant schedule and the predefined study period, 80 patients were ultimately enrolled. This limitation was considered in the interpretation of complication and DGF outcomes.

### Anesthetic protocol

All patients received a standardized anesthetic protocol. Premedication was not routinely administered. General anesthesia was induced with fentanyl, propofol, and rocuronium, then was maintained with continuous fentanyl infusion and desflurane titrated to a bispectral index (BIS) of 40–60. Mechanical ventilation was delivered in volume-controlled mode with a tidal volume of 8–10 mL/kg ideal body weight, positive end-expiratory pressure (PEEP) of 5 cm H_2_O, fraction of inspired oxygen (FiO_2_) of 0.6, respiratory rate of 12–14 breaths/min, and an end-tidal CO_2_ target of 35–45 mm Hg. Standard monitoring comprised of five-lead electrocardiography, noninvasive blood pressure, pulse oximetry, capnography, urinary output, temperature, and BIS. A central venous catheter and radial arterial line were placed in all patients. At the time of vascular anastomosis, all patients received furosemide 20–80 mg and methylprednisolone 250 mg intravenously.

### Fluid management protocol

Both groups received a baseline crystalloid infusion of Ringerfundin at 5 mL/kg/h from induction. The target mean arterial pressure (MAP) was ≥ 90 mm Hg during the pre-reperfusion phase. Intraoperative hypotension, defined as MAP < 65 mm Hg or a drop of > 20% from baseline, was treated with ephedrine 3 mg intravenous (IV) bolus. In the postoperative period, norepinephrine was titrated to maintain MAP ≥ 90 mm Hg if hypotension unrelated to hemorrhaged was identified.

In group C (CVP-guided), target CVP was 10–15 cm H_2_O. If CVP fell below 10 cm H_2_O, a crystalloid bolus of 200 mL was administered. If CVP was within the target range (10–15 cm H_2_O), maintenance infusion was continued at 5 mL/kg/h. Infusion was suspended if CVP exceeded 15 cm H_2_O.

In group P (PiCCO-guided GDHT), a 5F, 20-cm thermistor-tipped femoral arterial catheter was placed on the contralateral side to the transplant. Transpulmonary thermodilution calibration was performed by triple injection of cold saline (< 4 °C) via the central venous catheter. The PiCCO system continuously displayed SVV, PPV, CI, GEDVI, EVLWI, CFI, SVRI, and global ejection fraction (GEF). Fluid management decisions were guided by the following algorithm, with reassessment every 15 min throughout surgery:

- SVV/PPV 10–13%: continue maintenance infusion at 5 mL/kg/h.
- SVV/PPV > 13% (fluid-responsive): administer a 200 mL crystalloid bolus (maximum three sequential boluses); recalibrate thermodilution after each bolus.
- SVV/PPV < 10% (fluid-unresponsive): suspend infusion.

Additional hemodynamic targets monitored were CI > 2.5 L/min/m^2^, SVRI 1,700–2,400 dyn·s/cm^5^/m^2^, EVLWI < 10 mL/kg, and GEDVI within the reference range. A stepwise hypotension management algorithm (incorporating CI, GEDVI, SVV/PPV, EVLWI, GEF, CFI, SVRI, and hemoglobin concentration) guided the sequential use of fluid loading, dobutamine, noradrenaline, and blood transfusion when hemodynamic compromise occurred.

### Postoperative monitoring

All patients were extubated and managed in the post-anesthesia care unit. Urine output was recorded hourly. Serum creatinine was measured preoperatively and on postoperative days 1, 3, and 7. Renal and non-renal complications were documented during hospitalization. The primary outcome was early graft function, assessed by first-hour postoperative urine output and serum creatinine trajectory through day 7. Secondary outcomes were total intraoperative fluid volume, pre- and post-reperfusion fluid distribution, intraoperative hemodynamic stability, CVP, vasopressor use, EVLWI, and postoperative complications.

Renal complications were defined a priori as medical complications of the graft, principally DGF and acute rejection. DGF was defined as the need for dialysis within the first 7 days after transplantation. Additional graft-related events (graft pyelonephritis, urinary tract infection, peri transplant fluid collection, graft hematoma, transplant renal artery stenosis, and acute cystitis) were also recorded and classified as renal or non-renal complications.

### Monitoring timepoints

Hemodynamic variables were recorded at standardized timepoints: T0 (before induction), T1 (30 min post-induction), T2 (30 min pre-reperfusion), T3 (immediately post-reperfusion), and T4 (end of surgery).

### Statistical analysis

Statistical analysis was performed using STATA version 15.0 (StataCorp, College Station, TX, USA). Continuous data are expressed as mean ± standard deviation (SD) for normally distributed variables and as median (interquartile range) for non-normally distributed variables. Normality was quantitatively assessed using the Shapiro-Wilks test. Between-group comparisons were performed using the independent-samples Student’s *t*-test for parametric assumptions, or the Wilcoxon rank-sum test for non-parametric assumptions. Categorical variables were compared using the Chi-square test to assess for associations in a contingency table or Fisher’s exact test. A two-tailed P value of < 0.05 was considered statistically significant.

## Results

### Patient flow and baseline characteristics

Between May 2024 and December 2025, 80 living donor kidney transplant recipients were enrolled and consecutively assigned: 40 to group C and 40 to group P. All 80 patients completed the protocol and were included in the primary analysis; there were no withdrawals, protocol violations, or post-enrolment exclusions.

Baseline recipient, donor, and operative characteristics were largely comparable between groups (Table 1). Recipient age, sex distribution, dry weight, body mass index, donor age, ASA physical status, and operative time did not differ significantly between groups. Cold ischemia time was significantly longer in group P than in group C (120.9 ± 23.4 min vs. 108.2 ± 20.2 min; P = 0.011); this imbalance is addressed in the analysis and Discussion. Diabetes mellitus was recorded as a yes/no variable, and no recipient in either group had diabetes. All patients in both groups had a pre-existing diagnosis of hypertension (100%).

**Table 1:** Baseline Patient Characteristics

Characteristic	Group C (n = 40)	Group P (n = 40)	P value
Donor age (years), median (IQR)	54.5 (47–62)	53.0 (46–61)	0.725
Recipient age (years), mean ± SD	36.6 ± 7.7	36.4 ± 9.3	0.890
Male sex, n (%)	22 (55.0)	23 (57.5)	0.820
Dry weight (kg), mean ± SD	55.0 ± 9.8	54.5 ± 8.4	0.800
BMI (kg/m^2^), mean ± SD	20.9 ± 2.8	20.9 ± 2.2	0.970
Hypertension, n (%)	40 (100)	40 (100)	1.00
Diabetes, n (%)	0 (00)	0 (00)	1.00
ASA III/IV, n (%)	38 (95.0)/2 (5.0)	37 (92.5)/3 (7.5)	0.650
Cold ischemia time (min), mean ± SD	108.2 ± 20.2	120.9 ± 23.4	0.011
Operative time (min), mean ± SD	258.5 ± 46.3	249.4 ± 55.0	0.426

Continuous variables were compared using the two-sample *t*-test or Mann–Whitney U test, as appropriate; categorical variables were compared using the Fisher’s exact test when expected counts were sparse. BMI: body mass index; ASA: American Society of Anesthesiologists; IQR: interquartile range; SD: standard deviation.

### Graft function

Preoperative serum creatinine was comparable between groups (8.6 ± 2.4 vs. 8.9 ± 2.6 mg/dL; P = 0.60), confirming equivalent baseline renal disease severity (Table 2). On postoperative day 1, creatinine was similar in both groups (4.9 (3.1–6.2) vs. 4.6 (3.8–5.3) mg/dL; P = 0.47), reflecting the dominant early influence of ischemia–reperfusion injury. By postoperative day 3, serum creatinine was significantly lower in group P: 1.3 (0.8–1.8) vs. 1.6 (1.4–1.9) mg/dL, P = 0.036). On postoperative day 7, serum creatinine was numerically lower in group P, but the difference was not statistically significant (1.1 (0.7–1.4) mg/dL vs. 1.3 (1.1–2.1) mg/dL; P = 0.16).

**Table 2:** Graft Function

Variable	Group C (n = 40)	Group P (n = 40)	P value
Serum creatinine pre-op (mg/dL), mean ± SD	8.6 ± 2.4	8.9 ± 2.6	0.600
Serum creatinine day 1 (mg/dL), median (IQR)	4.6 (3.8–5.3)	4.9 (3.1–6.2)	0.470
Serum creatinine day 3 (mg/dL), median (IQR)	1.6 (1.4–1.9)	1.3 (0.8–1.8)	0.036
Serum creatinine day 7 (mg/dL), median (IQR)	1.3 (1.1–2.1)	1.1 (0.7–1.4)	0.16
Urine output Hour 1 post-op (mL), mean ± SD	1,010 ± 315	1,235 ± 162	< 0.001
Urine output day 1 (mL), mean ± SD	8,632 ± 2,477	9,023 ± 2,288	0.410
DGF (dialysis within 7 days), n (%)	0 (0)	0 (0)	1.000

Continuous variables were compared using the two-sample *t*-test or Mann–Whitney U test, as appropriate. IQR: interquartile range; SD: standard deviation; DGF: delayed graft function; post-op: postoperative.

Per protocol, all patients in both groups received furosemide 20 mg after reperfusion; no patient required an additional dose. First-hour postoperative urine output was significantly higher in group P (1,235 ± 162 mL vs. 1,010 ± 315 mL; P < 0.001). Total day 1 urine output was comparable (9,023 ± 2,288 vs. 8,632 ± 2,477 mL; P = 0.41). No patient in either group required dialysis within the first 7 postoperative days.

### Hemodynamics and vasopressor use

Intraoperative MAP was comparable between groups at all five timepoints (T0 through T4; all P > 0.05), confirming equivalent hemodynamic stability throughout the procedure (Table 3). CVP values were similarly comparable at all measured timepoints (T1 through T4; all P > 0.14).

**Table 3:** Intraoperative Hemodynamics

Variable	Group C (n = 40)	Group P (n = 40)	P value
MAP T0, before induction (mm Hg)	105.1 ± 15.5	103.2 ± 16.5	0.580
MAP T1, 30 min post-induction (mm Hg)	95.9 ± 14.7	94.0 ± 16.7	0.570
MAP T2, 30 min pre-reperfusion (mm Hg)	95.4 ± 15.9	94.3 ± 16.7	0.580
MAP T3, post-reperfusion (mm Hg)	102.0 ± 11.8	98.5 ± 11.0	0.140
MAP T4, End of surgery (mm Hg)	104.0 ± 10.9	102.2 ± 13.6	0.470
CVP T1 (cm H_2_O)	9.8 ± 2.8	9.2 ± 2.7	0.350
CVP T2 (cm H_2_O)	12.7 ± 3.8	14.1 ± 6.4	0.260
CVP T3 (cm H_2_O)	13.1 ± 3.1	14.4 ± 4.4	0.140
CVP T4 (cm H_2_O)	12.7 ± 3.0	13.0 ± 4.4	0.780
Vasopressor use, n (%)	11 (27.5)	5 (12.5)	0.161

Values are mean ± SD unless otherwise stated. Continuous variables were compared using the two-sample *t*-test or Mann–Whitney U test, as appropriate; categorical variables were compared using the Fisher’s exact test when expected counts were sparse. MAP: mean arterial pressure; CVP: central venous pressure.

Vasopressor use was numerically lower in group P than in group C (five patients (12.5%) vs. 11 patients (27.5%); this difference was not statistically significant (Fisher’s exact P = 0.161).

### Intraoperative fluid volumes

Total intraoperative fluid volume was significantly lower in group P than in group C (2,656 ± 480 mL vs. 3,097 ± 555 mL; P = 0.001), a mean reduction of 441 mL (Table 4). Pre-reperfusion fluid volume was also significantly lower in group P (1,871.7 ± 387.6 mL vs. 2,133.0 ± 436.5 mL; P = 0.006), as was post-reperfusion fluid volume (785.40 ± 343.73 mL vs. 964.00 ± 290.15 mL; P = 0.014). Thus, pre-reperfusion, post-reperfusion, and total intraoperative fluid volumes were all lower in the PiCCO-guided group, consistent with a more restrictive, individualized fluid strategy throughout surgery.

**Table 4:** Intraoperative Fluid Volumes

Variable	Group C (n = 40)	Group P (n = 40)	P value
Fluid pre-reperfusion (mL), mean ± SD	2,133.0 ± 436.5	1,871.7 ± 387.6	0.006
Fluid post-reperfusion (mL), mean ± SD	964,00 ± 290,15	785,40 ± 343,73	0.014
Total intraoperative fluid (mL), mean ± SD	3,097 ± 555	2,656 ± 480	0.001

Values are mean ± SD. Continuous variables were compared using the two-sample *t*-test or Mann–Whitney U test, as appropriate.

### PiCCO-derived parameters

In group P, SVV declined progressively from T1 to T2, reflecting volume optimization achieved by targeted fluid boluses prior to reperfusion (Table 5). SVV rose again at T4, consistent with post-reperfusion vasodilation and hemodynamic redistribution. EVLWI remained within the safe reference range (< 10 mL/kg) at T1 through T3, with a marginal increase at T4 (9.90 ± 4.9 mL/kg). No patient developed clinical or radiological evidence of pulmonary edema.

**Table 5:** PiCCO-Derived Parameters (Group P, N = 40)

Timepoint	T1, 30 min post-induction	T2, 30 min pre-reperfusion	T3, post-reperfusion	T4, end of surgery
SVV (%), mean ± SD	7.00 ± 2.9	4.53 ± 2.3	5.81 ± 3.9	7.65 ± 5.6
EVLWI (mL/kg), mean ± SD	8.75 ± 4.1	8.60 ± 4.1	8.87 ± 4.56	9.90 ± 4.9
EVLWI > 10 (mL/kg), n (%)	11 (27.5)	8 (20)	9 (22.5)	11 (27.5)
CI (L/min/m^2^), mean ± SD	3.21 ± 0.69	3.54 ± 0.79	3.92 ± 0.98	4.01 ± 1.02
GEDVI (mL/m^2^), mean ± SD	642 ± 110.1	661 ± 114.6	672 ± 117.1	698 ± 110.2
SVRI (dyn·s/cm^5^/m^2^), mean ± SD	2,277 ± 694.3	1,799 ± 653.1	1,769 ± 669.6	1,810 ± 449.6

Reference range EVLWI < 10 mL/kg. EVLWI > 10 (mL/kg), n (%) denotes the number of patients exceeding the 10 mL/kg threshold at each timepoint. All hemodynamic values are for the PiCCO group (group P) only; no between-group statistical comparison was performed. SVV: stroke volume variation; EVLWI: extravascular lung water index; CI: cardiac index (L/min/m^2^); GEDVI: global end-diastolic volume index (mL/m^2^); SVRI: systemic vascular resistance index (dyn·s/cm^5^/m^2^); PiCCO: pulse index continuous cardiac output.

CI, GEDVI, and SVRI in group P are reported by timepoint in Table 5. CI increased progressively from T1 to T4, whereas SVRI declined after reperfusion, consistent with the expected hemodynamic changes following graft reperfusion. Group mean EVLWI values remained within the reference range (< 10 mL/kg) throughout surgery, although the proportion of patients with EVLWI > 10 mL/kg ranged from 20.0% (T2) to 27.5% (T1 and T4). Although EVLWI exceeded 10 mL/kg in a subset of patients, GEDVI and other PiCCO-derived parameters were reviewed when interpreting volume status; no patient had EVLWI > 15 mL/kg, and no patient developed clinical or radiological evidence of pulmonary edema

### Postoperative complications

Renal complications occurred in two patients in group P (5.0%) and five patients in group C (12.5%; P = 0.23). Non-renal complications were identical between groups (three patients each, 7.5%; P = 1.00) (Table 6). Among renal complications, DGF and acute rejection were the principal events. These differences should be interpreted cautiously because the study was not powered to detect moderate differences in complication rates, and the findings should be considered exploratory and hypothesis-generating.

**Table 6:** Postoperative Complications

Complication	Group C (n = 40)	Group P (n = 40)	P value
Renal complications, n (%)	5 (12.5)	2 (5.0)	0.230
Non-renal complications, n (%)	3 (7.5)	3 (7.5)	1.000

Variables were compared using the Fisher’s exact test.

## Discussion

In this prospective observational cohort study of living donor kidney transplantation, PiCCO-guided GDHT was associated with lower total intraoperative fluid administration, numerically less frequent vasopressor use, higher first-hour postoperative urine output, and lower serum creatinine on postoperative days 3 and 7 compared with CVP-guided therapy. MAP and CVP were comparable between groups throughout surgery, suggesting that the observed differences were not attributable to gross differences in intraoperative pressure targets. These findings support the physiological rationale for individualized, dynamic hemodynamic management during kidney transplantation.

The lower total fluid volume in the PiCCO group likely reflects the use of SVV and PPV to identify when additional fluid was likely to increase stroke volume. In contrast, CVP-guided algorithms may lead to additional fluid administration despite limited fluid responsiveness because CVP does not reliably distinguish preload dependence from venous congestion or vasodilatory hypotension. The PiCCO protocol allowed fluid boluses when dynamic indices suggested responsiveness and suspension of infusion when dynamic indices suggested fluid unresponsiveness. This approach may reduce unnecessary volume loading while preserving perfusion pressure. In this study, pre-reperfusion, post-reperfusion, and total intraoperative fluid volumes were all lower in the PiCCO group, suggesting that the PiCCO protocol reduced fluid exposure throughout both phases of the operation. These findings are consistent with those of Fabes et al [20], Masri et al [24], and Cavaleri et al [19], all of whom reported lower intraoperative volumes with goal-directed strategies compared with standard care, without hemodynamic compromise.

The numerically lower vasopressor use in the PiCCO group, despite the lower total fluid volume, is clinically relevant. A multiparameter protocol can help differentiate low preload, impaired cardiac output, vasodilation, and potential fluid intolerance. This distinction is important in ESRD, where excessive fluid may increase venous congestion and pulmonary edema risk. Prior data linking intraoperative hypotension with DGF [6] emphasize the importance of maintaining graft perfusion, but the present findings suggest that adequate perfusion can be achieved without liberal fluid administration when dynamic and volumetric variables are available.

A key advantage of PiCCO over single dynamic variables is the concurrent assessment of EVLWI. In this study, EVLWI remained within the reference range throughout surgery, and no patient developed pulmonary edema. This observation is important because living donor kidney transplant recipients with ESRD may have chronic volume overload, impaired sodium and water excretion, and cardiovascular comorbidity. EVLWI may provide a safety boundary that is not available with CVP, PPV, or SVV alone [21].

The creatinine trajectory suggests faster early graft functional recovery in the PiCCO group. Serum creatinine was similar on postoperative day 1, which is consistent with the early influence of ischemia-reperfusion injury. A between-group difference was observed on postoperative day 3 (P = 0.036), whereas the day 7 difference was not statistically significant (P = 0.16); the median differences were small, and these findings are therefore interpreted as associative rather than mechanistic. No patient in either group developed DGF, consistent with a living donor cohort with relatively short ischemia times and good donor organ quality. Because DGF did not occur in either group, this outcome could not discriminate between the monitoring strategies, and early graft function was instead compared using first-hour urine output and the serum creatinine trajectory. Larger studies including higher-risk recipients will be needed to determine whether PiCCO-guided GDHT reduces DGF or improves longer-term graft survival.

These results are consistent with prior studies of goal-directed fluid therapy in kidney transplantation. Earlier randomized and observational studies using PPV or other dynamic parameter-guided strategies reported improved fluid balance and favorable postoperative outcomes compared with conventional care [19, 22, 23]. The present study extends this evidence by evaluating a multiparameter PiCCO-based protocol in a Southeast Asian transplant center, where evidence on advanced hemodynamic monitoring remains limited.

Several limitations should be acknowledged. First, this was a single-center observational cohort study with non-random allocation based on institutional practice, and residual confounding cannot be excluded. In particular, group allocation was driven by operating-room logistics. For PiCCO patients, donor nephrectomy and recipient transplantation were performed in separate operating room areas, and donor nephrectomy time varied by surgeon, whereas for CVP patients, the donor and recipient rooms were adjacent. These logistical differences did not balance cold ischemia time, which was significantly longer in group P; therefore, potential confounding from this imbalance cannot be excluded and should be considered in the interpretation of the results. Although vascular anatomy was similar between groups (14 recipients in group P had two or more renal arteries, including two with three arteries, versus 12 in group C, including one with three arteries; P = 0.63), the cold ischemia time imbalance and operating-room logistics remain potential sources of residual confounding. Second, the final sample size was smaller than planned, limiting statistical power for complications and DGF. Third, the study included only living donor recipients, which limits generalizability to deceased donor transplantation. Fourth, follow-up was limited to 7 postoperative days, preventing assessment of long-term graft function, rejection, graft survival, and patient survival. In addition, a small number of parameters requested during review were not captured in the study dataset and are acknowledged as limitations rather than reported. Specifically, group-level PTH, serum calcium, and serum phosphorus values were not available for analysis, and the fractional excretion of phosphate (FEp) and calcium (FECa) was not collected. Comorbidity and medication capture were restricted to variables recorded in the institutional dataset. Finally, the cost-effectiveness of PiCCO monitoring was not evaluated, which is important for implementation in resource-limited settings. Future randomized multicenter studies should include deceased donor recipients, longer follow-up, predefined cost-effectiveness analyses, and sample sizes powered for DGF and graft survival.

### Conclusions

PiCCO-guided GDHT was associated with reduced intraoperative fluid administration, numerically lower vasopressor use, higher first-hour postoperative urine output, and lower serum creatinine on postoperative days 3 in living donor kidney transplant recipients. These findings suggest that multiparameter dynamic hemodynamic monitoring may support individualized perioperative fluid management and early graft recovery. Randomized studies with longer follow-up are warranted to confirm these findings and determine their impact on DGF and long-term graft outcomes.

## Data Availability

The datasets generated and analyzed during the current study are available from the corresponding author upon reasonable request.

## Abbreviations

ASA: American Society of Anesthesiologists
BIS: bispectral index
BMI: body mass index
CI: cardiac index
CKD: chronic kidney disease
CVP: central venous pressure
DGF: delayed graft function
ESRD: end-stage renal disease
EVLWI: extravascular lung water index
FiO_2_: fraction of inspired oxygen
GDHT: goal-directed hemodynamic therapy
GEDVI: global end-diastolic volume index
IQR: interquartile range
MAP: mean arterial pressure
PEEP: positive end-expiratory pressure
PiCCO: pulse index continuous cardiac output
PPV: pulse pressure variation
SD: standard deviation
SVRI: systemic vascular resistance index
